# Immunohistochemical evaluation of a sarcomatoid hepatocellular carcinoma with osteoclastlike giant cells

**DOI:** 10.1186/s13000-015-0274-4

**Published:** 2015-04-28

**Authors:** Hans Helmut Dahm

**Affiliations:** Institut für Pathologie, Esslingen, Germany

**Keywords:** Liver, Heppar-1, Giant cell histiocytic reaction, Metaplastic carcinoma

## Abstract

**Background:**

Malignant liver tumors with osteoclast-like giant cells are rare. A literature search showed 17 previously reported cases that included the following: (1) 8 undifferentiated hepatocellular carcinomas, (2) 5 sarcomatous tumors with osteoclast-like giant cells associated with intrahepatic cholangiocarcinoma or liver cystadenocarcinoma, and (3) 4 sarcomatoid tumors with osteoclast-like giant cells associated with areas of a conventional hepatocellular carcinoma.

**Case presentation:**

A 68-year-old man presented with a tumor of the right lobe of the liver on ultrasonography and computed tomography. Laparoscopy showed a tumor (diameter, 4 cm) in segments 7 and 8 of the right liver lobe that adhered to the retroperitoneum. The tumor recurred 3 months after liver segmentectomy. Repeat laparoscopy showed diffuse and nodular metastases to the omentum and peritoneum.

**Result:**

Light microscopy showed that part of the tumor had features of classic hepatocellular carcinoma. Another part of the tumor had a solid sarcomatous pattern with osteoclast-like giant cells that were irregularly distributed between the smaller undifferentiated tumor cells; cells of this part of the tumor were positive for heppar-1.

**Conclusion:**

Light microscopic findings including osteoclast-like giant cells, and the strong reaction of heppar-1 antibody with cells of the sarcomatous part of the tumor, confirmed that this sarcomatous element was a metaplastic or transformed portion of hepatocellular carcinoma.

**Virtual Slides:**

The virtual slide(s) for this article can be found here: http://www.diagnosticpathology.diagnomx.eu/vs/6000512901462616

**Electronic supplementary material:**

The online version of this article (doi:10.1186/s13000-015-0274-4) contains supplementary material, which is available to authorized users.

## Background

Malignant liver tumors with osteoclast-like giant cells are extremely rare. Since this entity was described in 1980, a total of 17 cases have been reported. In 8 of these tumors, histology showed similarities to giant cell tumor of bone, including multiple osteoclast-like giant cells and proliferating malignant undifferentiated mononuclear cells [1-8]. The clinical appearance of these tumors might be consistent with an unusual variant of hepatocellular carcinoma. According to the World Health Organization classification of tumors of the digestive system, these tumors might be classified as undifferentiated carcinomas of the liver [9]. In 5 of the 17 tumors, the sarcomatous tumors with osteoclast-like giant cells were associated with intrahepatic cholangiocarcinomas or cystadenocarcinomas of the liver [10-14]. Only 4 of the 17 tumors that have been reported could be classified as sarcomatoid hepatocellular carcinomas with osteoclast-like giant cells, according to the World Health Organization classification, because they comprised sarcomatoid tumors with osteoclast-like giant cells and areas of classic hepatocellular carcinoma. In 2 of these 4 tumors, transitional features were noted between the conventional hepatocellular carcinoma and the sarcomatous portion of the tumor that contained osteoclast-like giant cells [15-18].

The histogenesis of the sarcomatoid portion of these liver tumors is controversial because of the similarity to giant cell tumor of bone or other mesenchymal tumors that have osteoclast-like giant cells. Literature search showed no previous reports of sarcomatoid hepatocellular carcinoma with osteoclast-like giant cells in which the sarcomatous tumor could be described unequivocally as a transformed or metaplastic portion of classic liver cell carcinoma based on morphology or additional immunohistochemical studies including reactivity with heppar-1 antibody. We evaluated a patient who had sarcomatoid hepatocellular carcinoma that was associated with osteoclast-like giant cells.

## Case presentation

### Clinical evaluation

A 68-year-old man was suspected to have a tumor of the right lobe of the liver based on ultrasonography that was performed during a routine examination. A computed tomography (CT) scan showed evidence of a malignant liver neoplasm. The past medical history included insulin-dependent type 2 diabetes mellitus and hypertension for 10 years. The patient had a long history of consuming 2 L wine and cider daily. Laboratory findings showed slightly increased levels of gamma-glutamyl transferase, liver transaminases, carcinoembryonic antigen, and carbohydrate antigen 19–9. Antihepatitis B core and antihepatitis C virus tests were negative. Laparoscopy showed a tumor (diameter, 4 cm) in segments 7 and 8 of the right liver lobe that adhered to the retroperitoneum. Biopsies of the tumor and tumor-free liver tissue were sent for frozen section analyses. Additional staging procedures were negative for bone and lung metastases. The patient requested surgery, and atypical segmentectomy of the liver was performed 2 weeks later. Spiral CT scan of the abdomen 3 months later showed tumor recurrence in segment 6 of the right lobe of the liver. The patient developed massive ascites. Repeat laparoscopy showed diffuse and nodular metastases to the omentum and peritoneum of the right upper abdomen. Tumor biopsies were sent for frozen section analysis. Palliative therapy was recommended.

## Method

The tissue specimens were fixed in 10% formalin and embedded in paraffin. Routine stains and immunostains were performed on 4-μm sections. The following antibodies were applied: antihuman hepatocyte (Heppar-1, clone OCH1E5, 1:200, Dako Denmark, Glostrup, Denmark), CD68 (clone KP1, 1:3000, Dako), acid phosphatase (clone 26E5, 1:50, Novocastra, Newcastle upon Tyne, UK), and cytokeratin (AE1/AE3, 1:100, Zytomed Systems, Berlin, Germany). The deparaffinized and rehydrated sections were subjected to heat-induced epitope retrieval with citrate buffer (pH, 6.0) at a dilution of 1:10 (Zytomed) in an oven (96°C). Staining was performed in an automated stainer (Dako Autostainer, Dako). A streptavidin-biotin kit (ZytoChem-Plus HRP Kit, Broad Spectrum, Zytomed) was used for detection and combined with a 3,3′-diaminobenzidine tetrahydrochloride chromogen (DAB substrate kit, Zytomed).

### Gross examination

**Figure 1:**
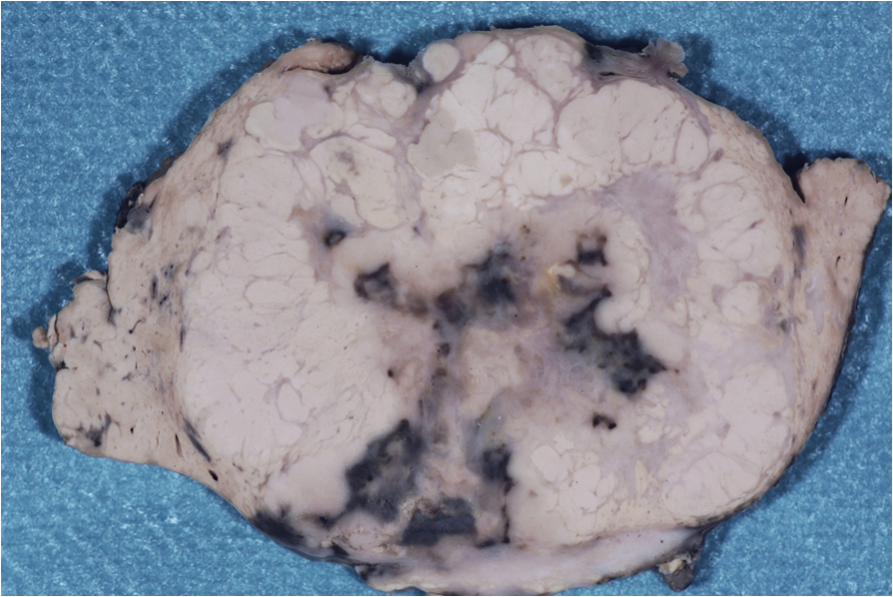
Multinodular tumor of the liver with central necrosis and hemorrhage surrounded by normal liver tissue.

At the first diagnostic laparoscopy, a wedge liver biopsy was performed (8 × 5 × 3 mm) and additional smaller pieces were excised. The atypical segmentectomy specimen (9 × 8 × 5 cm; 115 g) contained a tumor (largest diameter, 6 cm) that was well demarcated and surrounded by normal yellow and brown liver parenchyma. The gray-white tumor included multiple confluent nodules of various sizes and contained areas of fresh and old hemorrhage and fibrosis (Figure 1).

### Histology

**Figure 2:**
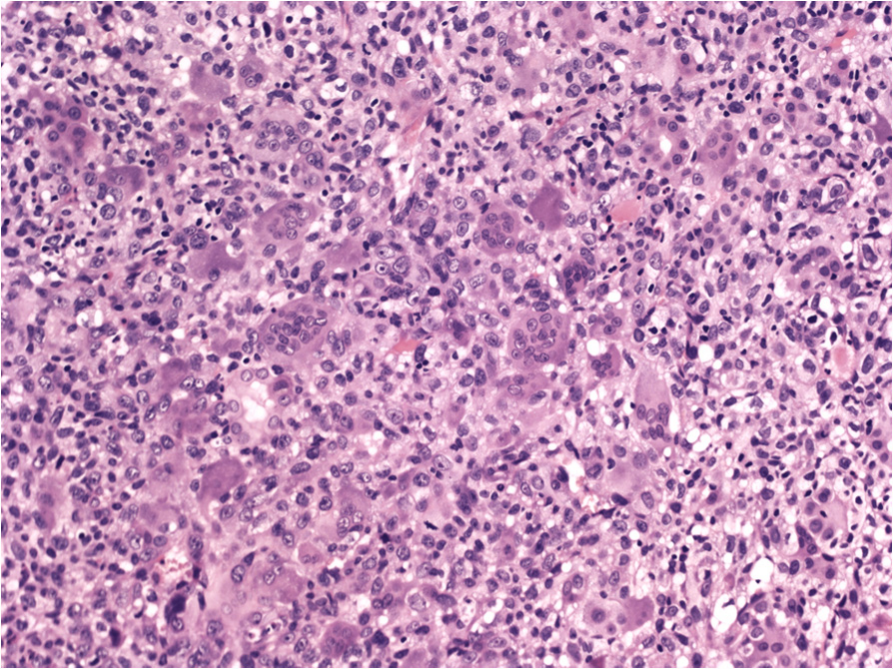
Sarcomatoid hepatocellular carcinoma with osteoclast-like giant cells and a minimal epithelial component (hematoxylin-eosin, original magnification × 200).

The tumor-free parenchyma of the liver had marked chronic periportal hepatitis and moderate fibrosis, residual features of alcoholic hepatitis that were noted in the previous biopsy. There was no high-grade fibrosis or cirrhosis of the liver. The first biopsy of the liver tumor showed carcinoma with multiple osteoclast-like giant cells (Figure 2). The possibility of a secondary tumor could not be excluded.

**Figure 3:**
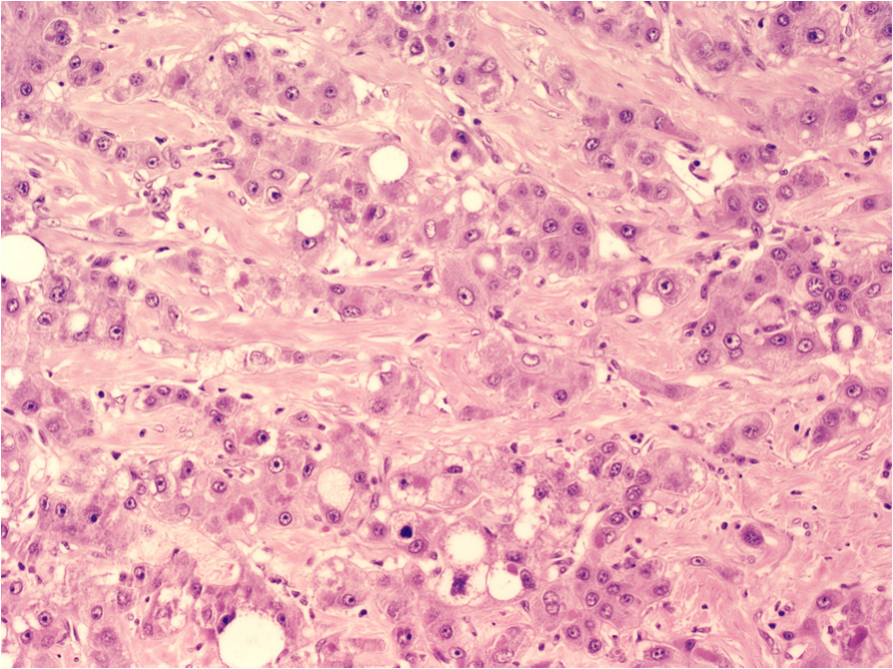
Scirrhous growth pattern of the hepatocellular carcinoma that resembled a fibrolamellar carcinoma (hematoxylin-eosin, original magnification × 200).

Light microscopic examination showed that the tumor in the atypical liver resection specimen was incompletely encapsulated. It was composed of multiple nodules of different sizes that were incompletely encapsulated by fibrous bands of varied thickness. The microscopic appearance of the tumor was highly varied in the different nodules. Part of the tumor exhibited features of classic hepatocellular carcinoma, and another part of the tumor exhibited a solid sarcomatous pattern. The hepatocellular carcinoma contained a large variety of growth patterns that included trabecular and pseudoglandular or compact patterns. Most of the atypical hepatocytes of these well-to-moderately-differentiated areas of the carcinoma had large, well-delineated granular eosinophilic cytoplasm and moderately enlarged nuclei with prominent nucleoli. In the less-well-differentiated areas, the cells contained bizarre hyperchromatic giant nuclei. The cytoplasm of numerous atypical hepatocytes contained Mallory bodies, eosinophilic bodies, and fat. The supporting connective tissue was inconspicuous in most parts of the tumor. In a few areas, a scirrhous growth pattern was noted with broad bands of hyalinized collagen fibers along the sinusoids that resembled fibrolamellar carcinoma (Figure 3). Additionally, there were sparse areas of carcinoma with duct-like features that were similar to bile duct proliferation in the adjacent scar tissue.

**Figure 4:**
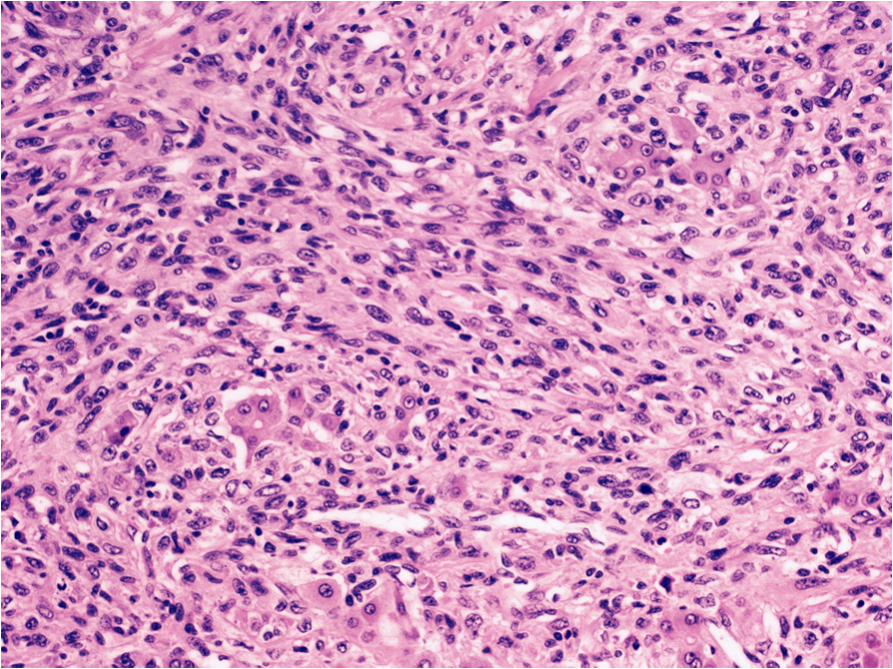
Sarcomatoid hepatocellular carcinoma with features of a spindle-cell sarcoma and a minimal epithelial component (hematoxylin-eosin, original magnification × 200).

The sarcomatous portion of the liver tumor included polymorphous, spindle-shaped, or oval cells (small-to-moderate size) with small, ill-defined clear cytoplasm. The nuclei were round or spindle-shaped with overall marked anaplasia. Blast-like cells had large vesicular nuclei and prominent nucleoli. Mitotic figures were noted frequently. The spindle-shaped tumor cells occasionally formed broad fascicles that had the appearance of an undifferentiated spindle cell sarcoma. In some nodules, these spindle cell proliferations enclosed trabeculae of atypical liver cells or tubular structures (Figure 4).

In the sarcomatous portion of the tumor, larger areas were noted that had osteoclast-like giant cells that were irregularly distributed between the smaller undifferentiated tumor cells. In these areas, the tumor resembled giant cell tumor of bone. The osteoclast-like giant cells had a large eosinophilic cytoplasm and contained many small round nuclei with small nucleoli. In a few of the nodules, the tumor consisted of a mixture of osteoclast-like giant cells and small syncytial complexes and tubules of atypical epithelial cells. Both components of these nodules appeared very similar and might have been mixed with each other in the hematoxylin-eosin stain. The metastatic tumor of the peritoneal biopsies had essential features of the primary tumor including osteoclast-like giant cells.

### Immunohistochemistry

**Figure 5:**
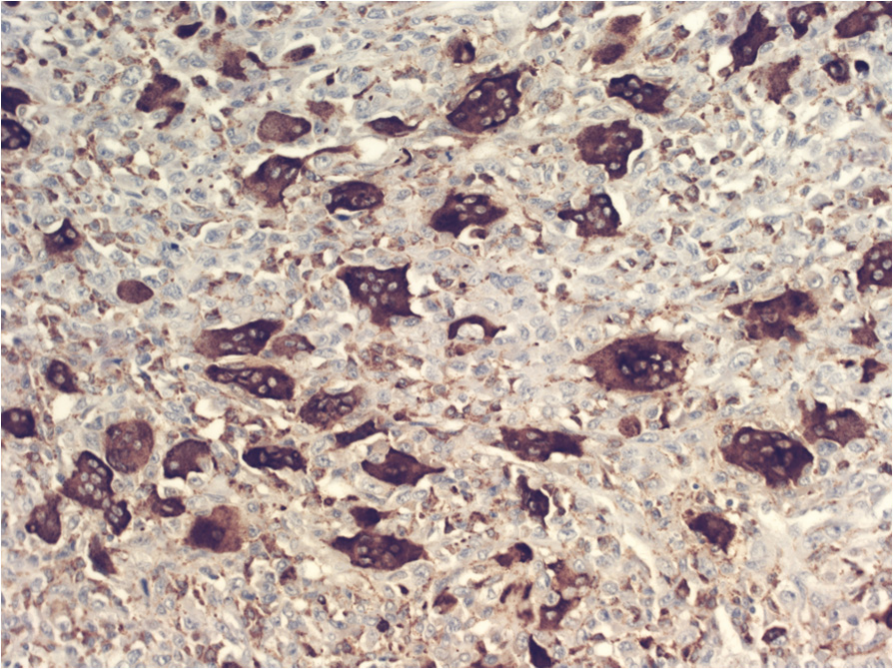
CD68 staining of the osteoclast-like giant cells distributed among atypical mononuclear undifferentiated cells and epithelial cells (original magnification × 200).

The antibody heppar-1 strongly reacted with the tumor-free liver tissue and the tumor cells of the well-to-moderately-differentiated hepatocellular carcinoma. The staining of the areas of the less-well-differentiated carcinoma with marked nuclear anaplasia was decreased or patchy. The sarcomatous portion of liver tumor was almost completely negative for heppar-1 and cytokeratin AE1/AE3. The osteoclast-like giant cells and small mononuclear histiocytic cells of the tumor stroma were positive for the histiocytic markers CD68 and acid phosphatase (Figure 5) and were cytokeratin AE1/AE3-negative. The sparse areas with duct-like features were positive for cytokeratin AE1/AE3 and focally positive for heppar-1.

**Figure 6:**
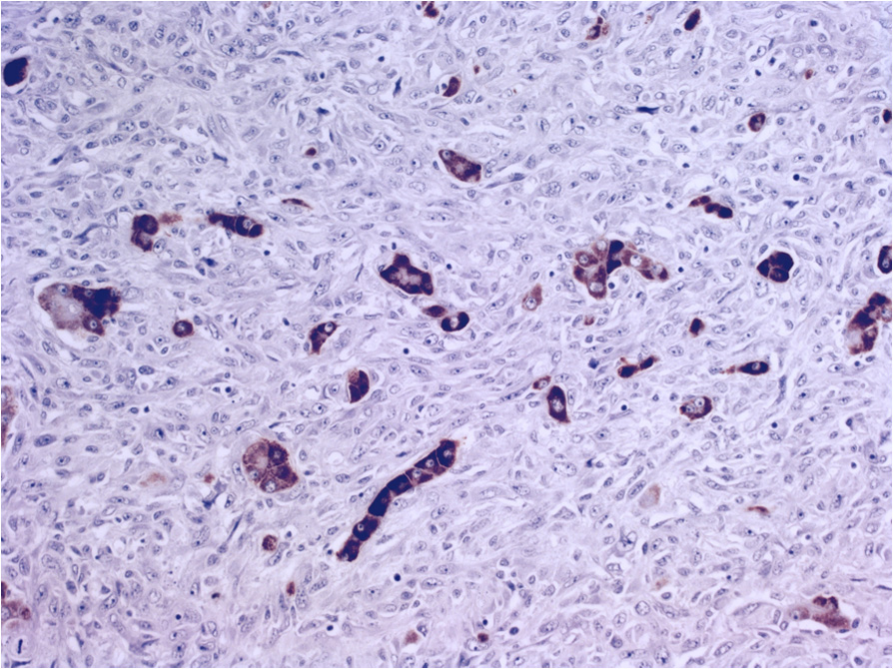
The heppar-1 stain marked the preserved isolated atypical hepatocytes and tubules in the sarcomatoid stroma (original magnification × 200).

**Figure 7:**
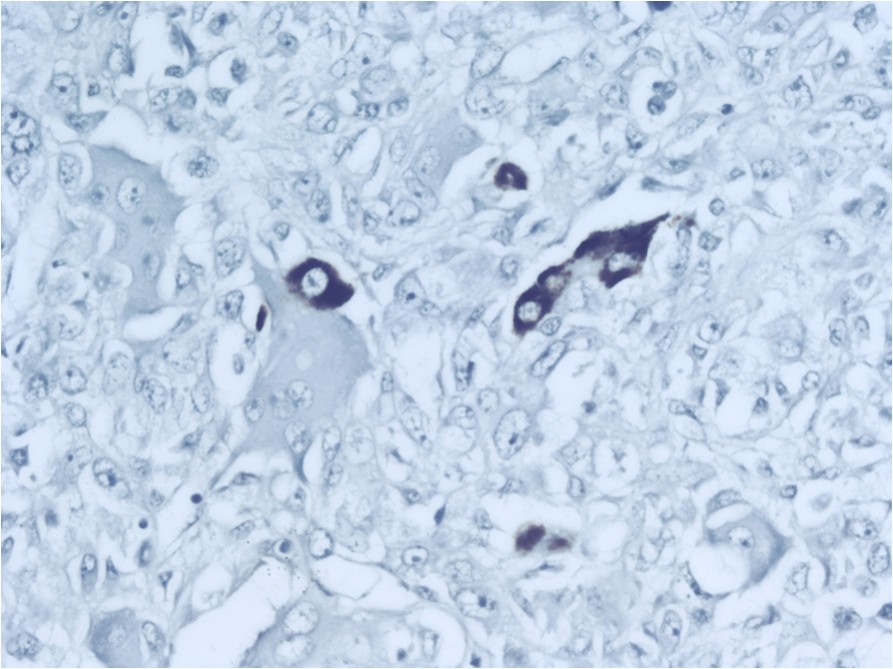
Isolated heppar-1-positive tumor cell bordering an osteoclast-like giant cell (original magnification × 400).

The tumor area with the scirrhous pattern was CD68-negative. In the areas in which the osteoclast-like giant cells and epithelial cell complexes were intensely mixed, the CD68 stain (unlike the hematoxylin-eosin stain) distinguished the 2 cell types; the epithelial tumor cells remained unstained and were marked only by the counterstain. However, a few malignant epithelial cell proliferations in these areas were positive for heppar-1 and AE1/AE3. The classic hepatocellular carcinoma and the area of sarcomatous growth exhibited occasional areas of confluence. In the other areas, the epithelial tumor was infiltrated or destroyed by sarcomatous tumor growth. The close contact of tissue with both growth patterns mimicked transformation of the epithelial tumor to a sarcomatous growth pattern. However, the full appearance of transformation became visible at higher magnification in other areas in which the trabeculae of the atypical hepatocytes of the conventional hepatocellular carcinoma (which exhibited large eosinophilic cytoplasm and marked nuclear anaplasia) transformed into strands and nests of smaller cells (that exhibited ill-defined clear cytoplasm). The atypical liver cells acquired the characteristics of the surrounding sarcomatous tumor cells but occasionally could be diagnosed as hepatocytes because of their Mallory body content. This impression was supported by findings of strongly positive heppar-1 staining in the single epithelial cells and trabeculae or small tubules of the epithelial tumor cells within the sarcomatous tumor tissue (Figure 6). Very rarely, heppar-1-positive single epithelial tumor cells were observed in close contact with the osteoclast-like giant cells (Figure 7).

## Discussion

This 68-year-old man in whom a liver tumor was noted on ultrasonography and CT scan received a diagnosis of carcinoma with osteoclast-like giant cells based on analyses of laparoscopic frozen sections. The atypical liver resection specimen contained a tumor (diameter, 6 cm), and there were no signs of cirrhosis in the tumor-free liver tissue. Histologic evaluation of the liver tumor showed 2 parts that included conventional hepatocellular carcinoma and a sarcomatous tumor with multiple osteoclast-like giant cells. The hepatocellular carcinoma had multiple growth patterns that included trabecular, pseudoglandular, and compact patterns. A few areas had the tubular features that can occur in 5% to 10% trabecular liver cell carcinomas [11].

The fibrotic areas of the tumor also had the appearance of fibrolamellar carcinoma; however, fibrolamellar carcinomas are CD68-positive [19], and the fibrotic area in the present tumor was CD68-negative. Therefore, the present tumor was classified as having the scirrhous growth pattern of hepatocellular carcinoma [20].

The transformation of classic liver cell carcinoma to the sarcomatous tumor was observed at higher magnification with hematoxylin-eosin stain where the trabeculae of the atypical hepatocytes blended into strands and nests of small tumor cells. These small tumor cells had ill-defined clear cytoplasm, and became indistinguishable from the cells of the surrounding sarcomatous tumor tissue by light microscopy. These undifferentiated cells occasionally contained Mallory bodies, which is consistent with a tumor of liver cell origin. The strongly positive heppar-1 staining of the single cells and trabeculae of the epithelial cells and tubules in the sarcomatous component of the liver tumor provided further evidence of transformation of conventional hepatocellular carcinoma into a sarcomatous tumor. The transition of trabecular hepatocellular carcinoma to spindle cell hepatocellular carcinoma has been well documented for sarcomatoid hepatocellular carcinomas [11]. However, we were unable to find any previous reports that confirmed this transition in a sarcomatoid hepatocellular carcinoma with osteoclast-like giant cells by immunohistochemical methods.

A literature search identified 17 previously reported liver tumors with osteoclast-like giant cells that resembled an osteoclastoma or giant cell tumor of bone. There were 3 histologic types in these previously reported tumors: (1) undifferentiated tumors (8 tumors) that were not associated with conventional carcinomas, (2) sarcomatous tumors (5 tumors) that were associated with a cholangiocarcinoma or cystadenocarcinoma of the liver, and (3) sarcomatous tumors that were associated with classic hepatocellular carcinomas (4 tumors), as observed in the present patient.

The first osteoclastoma-like giant cell tumor of the liver with a tumor stroma of malignant mononuclear cells with polygonal or spindle-shaped nuclei was described in 1980 [6]. Numerous osteoclast-like giant cells were distributed among the pleomorphic mononuclear cells. The absence of hepatocellular carcinomatous features was noted. A transition of the hepatocytes to tumor cells from the disrupted hepatic cell plates was not observed. There were no ultrastructural features that indicated an epithelial or hepatocellular origin of the malignant mononuclear cells. However, the presentation, clinical course, and gross pathology closely resembled hepatocellular carcinoma [6].

Another cellular liver tumor had 2 completely different cell types (osteoclast-like giant cells and pleomorphic mononuclear cells). Again, no histologic features typical of hepatocellular carcinoma were observed. The tumor was negative for keratin. Electron microscopic observation of desmosome-like junctions suggested the diagnosis of spindle cell carcinoma [2].

The same results were observed in a third tumor of the liver with similar features [3]. Another example of this tumor type included mononuclear tumor cells that stained strongly positive for alpha-fetoprotein and weakly positive for anticytokeratin (CAM 5.2) [5]. The osteoclast-like giant cells were strongly positive for CD68 staining. No epithelial features were identified on electron microscopy.

Another liver tumor contained no overt hepatocellular carcinoma but exhibited features reminiscent of hepatocellular carcinoma in a small region that contained round cohesive tumor cells in a vaguely trabecular pattern [8]. A few malignant cells were positive for cytokeratins (CK7, CK8, and CK19). In another similar tumor, no trabecular or lobular pattern was noted. The focal positivity for CK19 and epithelial membrane antigens supported the diagnosis of undifferentiated carcinoma of the liver with an abundant osteoclast-like giant cell component [1]. Another tumor that was diagnosed as an osteoclast-like giant cell tumor of the liver contained malignant cells that were immunohistochemically positive for alpha-1-antitrypsin and alpha-1-chymotrypsin but negative for epithelial markers [7].

The origin of 1 carcinoma with osteoclast-like giant cells was documented from the wall of a large cyst of the liver [11]. This tumor was similar to a previously described tumor [6], and might be included in the group of cholangiocarcinomas. A sarcomatoid cholangiocellular carcinoma with osteoclast-like giant cells has been described with apparent tubule formations and predominant sarcomatous features. The spindle-shaped malignant cells exhibited positive reactions to cytokeratin and vimentin [12].

An osteoclast-like giant cell tumor of the liver that was associated with atypical intraductal epithelial proliferations was evaluated for mutations at codon 12 of the K-ras oncogene to determine the relation between the noninvasive epithelial proliferations and the infiltrating tumor. The identical genetic findings suggested that the osteoclast-like giant cell tumor of the liver was an undifferentiated cholangiocarcinoma [14].

In another report, the authors described the association of a mucoepidermoid variant of a cholangiocarcinoma of the liver and an osteoclast-like giant cell tumor [10]. Another liver tumor with osteoclast-like giant cells presented as a thrombus in the inferior vena cava; this tumor was diagnosed as an undifferentiated cholangiocellular carcinoma on the basis of strong positivity of the tumor cells for CK19 and CK7 [13]. Similar findings were described in tumors of type 1; therefore, although the tumors of this type were interpreted as hepatocellular carcinomas, some of them might also have been undifferentiated cholangiocarcinomas or this tumor might be included in type 1.

The sarcomatoid hepatocellular carcinomas (type 3), including the present tumor, are characterized by the combined occurrence of conventional hepatocellular carcinoma and sarcomatous tumor with osteoclast-like giant cells. In 1 of these tumors, 2 distinct areas included a conventional hepatocellular carcinoma with a trabecular pattern and an osteoclast-like giant cell tumor, separated by connective fibrous tissue [15].

A previously described and similar liver tumor exhibited several microscopic fields, and < 5% of the tumor had the histologic appearance of typical hepatocellular carcinoma [16]. The diagnosis was hepatic giant cell carcinoma. This latter term was used by others to designate primary liver tumors with either multinucleated or pleomorphic large cells [11,16]. Other workers have suggested that the term *giant cell carcinoma* in this case might be misleading and that the term *liver cell carcinoma with osteoclast-like giant cells* might be more accurate [21]. In a third report, a multifocal primary liver tumor was diagnosed as moderately differentiated hepatocellular carcinoma with a trabecular pattern in segment 4 and poorly differentiated hepatocellular carcinoma with a sarcomatoid pattern with osteoclast-like giant cells in segments 7 and 8 [17]. The fourth reported tumor of this type had 2 distinct components: a well-to-moderately differentiated conventional hepatocellular carcinoma with trabecular and pseudoglandular growth patterns, and a tumor with spindle-to-oval mononuclear cells and osteoclast-like giant cells [18]. There were transitions between both components in a single nodule. The 4 cited articles described liver tumors that had classic hepatocellular carcinomas and areas of sarcomatous tumors with osteoclast-like giant cells [15-18]. The authors of 2 of these cases concluded from the close proximity of the carcinomatous and sarcomatous tumor elements that the latter might have been a dedifferentiated part of the liver cell carcinoma [17,18]. The present case provides additional evidence for a transition from a carcinomatous to sarcomatous tumor; based on hematoxylin-eosin staining and immunohistochemical evaluation with heppar-1 antibody, the sarcomatous element of the liver tumor probably was a metaplastic hepatocellular carcinoma. Tumors that contain scattered multinucleated giant cells comparable to normal osteoclasts have been described in varied sites including the thyroid, lung, breast, pancreas, and salivary glands [21]. They mostly are interpreted as rare neoplasms composed of highly pleomorphic neoplastic mononuclear cells and large, nonneoplastic multinucleated, histiocytic giant cells [22]. This interpretation was favored in most articles about liver tumors discussed above, on the basis of the histologic and immunohistochemical findings. However, in 1 case, both the osteoclast-like giant cells and mononuclear cells were considered mesenchymal in origin [6]. In another liver tumor, both mononuclear and giant cells showed ultrastructural features typical of hepatocellular carcinoma [16].

The nonneoplastic multinucleated giant cells are termed *osteoclast-like* because of their resemblance to osteoclasts. On scanning electron microscopy, the 2 cell types are indistinguishable. They also share some features by transmission electron microscopy, but the osteoclast-like cells lack the characteristic complex ruffled border of the osteoclast. The subplasmalemmal accumulation of polyribosomes and cisternae of the rough endoplasmic reticulum of the osteoclast-like cells marks them as histiocytic cells. In tissue culture, the osteoclast-like cells can resorb bone, similar to osteoclasts. This suggests that this function of these macrophage-derived cells is induced by local factors, which also may explain giant cell formation [23]. Therefore, this type of carcinoma may have the capacity to induce nonneoplastic, osteoclast-like elements within themselves [21]. At present, the occurrence of these osteoclast-like giant cells in very few carcinomas at different sites remains an enigma.

## Conclusion

The present sarcomatoid hepatocellular carcinoma with osteoclast-like giant cells is a rare tumor of the liver, and only 4 other comparable cases have been described previously. These tumors have areas of classic liver cell carcinoma and a sarcomatous tumor with multiple osteoclast-like giant cells. The liver cell carcinoma portion of the present tumor was remarkable because it combined several rare growth patterns. Additionally, the transformation from epithelial to mesenchymal tumor was shown with routine stains and immunohistochemical evaluation, and this confirmed that the sarcomatous portion of the tumor with osteoclast-like giant cells represented the metaplastic part of the hepatocellular carcinoma. Analogous features might be observed in similar tumors from different sites.

## Consent

Written informed consent was obtained from the next of kin of the patient for publication of this Case Report and any accompanying images. A copy of the written consent is available for review by the Editor-in-Chief of this journal.
